# Association of Neighborhood Disadvantage in Childhood With DNA Methylation in Young Adulthood

**DOI:** 10.1001/jamanetworkopen.2020.6095

**Published:** 2020-06-01

**Authors:** Aaron Reuben, Karen Sugden, Louise Arseneault, David L. Corcoran, Andrea Danese, Helen L. Fisher, Terrie E. Moffitt, Joanne B. Newbury, Candice Odgers, Joey Prinz, Line J. H. Rasmussen, Ben Williams, Jonathan Mill, Avshalom Caspi

**Affiliations:** 1 Department of Psychology and Neuroscience, Duke University, Durham, North Carolina; 2 Social, Genetic, and Developmental Psychiatry Centre, Institute of Psychiatry, Psychology & Neuroscience, King’s College London, London, United Kingdom; 3 Center for Genomic and Computational Biology, Duke University, Durham, North Carolina; 4 Institute of Psychiatry, Psychology & Neuroscience, Department of Child & Adolescent Psychiatry, King’s College London, London, United Kingdom; 5 National and Specialist Child and Adolescent Mental Health Service Clinic for Trauma, Anxiety, and Depression, South London and Maudsley National Health Service Foundation Trust, London, United Kingdom; 6 Department of Psychiatry and Behavioral Sciences, Duke University, Durham, North Carolina; 7 Sanford School of Public Policy, Duke University, Durham, North Carolina; 8 Department of Psychological Science, University of California, Irvine, Irvine; 9 Clinical Research Centre, Copenhagen University Hospital Amager and Hvidovre, Hvidovre, Denmark; 10 University of Exeter Medical School, University of Exeter, Exeter, United Kingdom

## Abstract

**Importance:**

DNA methylation has been proposed as an epigenetic mechanism by which the childhood neighborhood environment may have implications for the genome that compromise adult health.

**Objective:**

To ascertain whether childhood neighborhood socioeconomic disadvantage is associated with differences in DNA methylation by age 18 years.

**Design, Setting, and Participants:**

This longitudinal cohort study analyzed data from the Environmental Risk (E-Risk) Longitudinal Twin Study, a nationally representative birth cohort of children born between 1994 and 1995 in England and Wales and followed up from age 5 to 18 years. Data analysis was performed from March 15, 2019, to June 30, 2019.

**Exposures:**

High-resolution neighborhood data (indexing deprivation, dilapidation, disconnection, and dangerousness) collected across childhood.

**Main Outcomes and Measures:**

DNA methylation in whole blood was drawn at age 18 years. Associations between neighborhood socioeconomic disadvantage and methylation were tested using 3 prespecified approaches: (1) testing probes annotated to candidate genes involved in biological responses to growing up in socioeconomically disadvantaged neighborhoods and investigated in previous epigenetic research (stress reactivity–related and inflammation-related genes), (2) polyepigenetic scores indexing differential methylation in phenotypes associated with growing up in disadvantaged neighborhoods (obesity, inflammation, and smoking), and (3) a theory-free epigenome-wide association study.

**Results:**

A total of 1619 participants (806 female individuals [50%]) had complete neighborhood and DNA methylation data. Children raised in socioeconomically disadvantaged neighborhoods exhibited differential DNA methylation in genes involved in inflammation (β = 0.12; 95% CI, 0.06-0.19; *P* < .001) and smoking (β = 0.18; 95% CI, 0.11-0.25; *P* < .001) but not obesity (β = 0.05; 95% CI, −0.01 to 0.11; *P* = .12). An epigenome-wide association study identified multiple CpG sites at an arraywide significance level of *P* < 1.16 × 10^−7^ in genes involved in the metabolism of hydrocarbons. Associations between neighborhood disadvantage and methylation were small but robust to family-level socioeconomic factors and to individual-level tobacco smoking.

**Conclusions and Relevance:**

Children raised in more socioeconomically disadvantaged neighborhoods appeared to enter young adulthood epigenetically distinct from their less disadvantaged peers. This finding suggests that epigenetic regulation may be a mechanism by which the childhood neighborhood environment alters adult health.

## Key Points

**Question:**

Is childhood neighborhood disadvantage associated with differential DNA methylation?

**Findings:**

In this cohort study of 1619 children in Great Britain, exposure to neighborhood socioeconomic disadvantage during childhood was associated with differential DNA methylation at age 18 years in genes involved in inflammation, exposure to tobacco smoke, and metabolism of toxic air pollutants.

**Meaning:**

The study found that children who were raised in socioeconomically disadvantaged neighborhoods appeared to enter young adulthood epigenetically distinct from their more advantaged peers.

## Teaser

This cohort study traces the biological responses and associated phenotypes of an upbringing in a socially and economically disadvantaged environment.

## Introduction

Children raised in socioeconomically disadvantaged neighborhoods grow up to have worse health as adults compared with their peers from more affluent communities,^1,2,3^ a phenomenon not fully explained by individual- or family-level socioeconomic factors or by the self-selection of families with more illness to move into poorer neighborhoods.^4,5,6^ Environmentally induced alterations to the epigenome have been proposed as one potential mechanism linking early-life neighborhood environments to later-life disease and dysfunction.^7,8^ Although previous studies have reported an association between individual-level socioeconomic factors and differential DNA methylation patterns,^9,10,11,12^ only a handful have evaluated whether characteristics of the wider neighborhood environment demonstrate a corresponding, and independent, association with epigenetic differences.

To our knowledge, 7 studies have tested for DNA methylation differences among individuals living along neighborhood socioeconomic gradients (eTable 1 in the Supplement).^13,14,15,16,17,18,19^ Each study reported associations between measured neighborhood characteristics and some DNA methylation targets, supporting the premise that the neighborhood environment may have implications for the epigenome. These studies are not without limitations, however.^14^ First, some were underpowered to detect subtle associations; of the 7 studies, 5 had fewer than 250 participants. Second, most quantified DNA methylation at sites that collectively represent only a small subset of potential targets. Third, none was able to rule out the possibility that methylation differences resulted from the proximal behaviors (eg, smoking) or conditions (eg, obesity) that characterize individuals living in socioeconomically disadvantaged neighborhoods.

In this cohort study, we sought to replicate and expand the initial reports about neighborhood characteristics and DNA methylation using data from participants in the Environmental Risk (E-Risk) Longitudinal Twin Study, a nationally representative birth cohort of same-sex twins born between 1994 and 1995 in England and Wales and followed up to age 18 years (through September 2014).^20^ The E-Risk Study cohort included ample numbers of children growing up in Britain’s most disadvantaged local areas. We measured multiple aspects of the participants’ neighborhoods across childhood and adolescence, indexing neighborhood deprivation, dilapidation, disconnection, and dangerousness. We then integrated neighborhood assessments with measures of DNA methylation in whole blood drawn at age 18 years to test the hypothesis that children raised in more socioeconomically disadvantaged neighborhoods show differential methylation patterns in young adulthood compared with their peers raised in more advantaged neighborhoods.

We preregistered 3 distinct approaches to studying the associations between neighborhood socioeconomic disadvantages and methylation: (1) methylation of probes that were annotated to candidate genes putatively involved in biological responses to growing up in disadvantaged environments (ie, stress reactivity–related and inflammation-related genes),^18^ (2) methylation of probes known to be differentially methylated in phenotypes associated with growing up in socioeconomically disadvantaged environments (ie, obesity, inflammation, and smoking), and (3) methylation of probes identified through an epigenome-wide association study (EWAS) of the association between neighborhood disadvantage and quantitative methylation measured at approximately 430 000 CpG sites on the Illumina 450k methylation assay (Infinium HumanMethylation450 BeadChip; Illumina, Inc).

## Methods

The Joint South London and Maudsley and the Institute of Psychiatry Research Ethics Committee approved each phase of the E-Risk Study. Parents gave written informed consent, and the twins gave assent at age 5 to 12 years and then informed consent at age 18 years. Further details are reported elsewhere^20^ and in the eAppendix 1 in the Supplement. We followed the Strengthening the Reporting of Observational Studies in Epidemiology (STROBE) reporting guideline.

### E-Risk Study Cohort

The current study uses a sample of the E-Risk Study cohort with complete DNA methylation data. Participants are members of the E-Risk Study, which tracked the development of a nationally representative birth cohort of 2232 twin children born between 1994 and 1995 in England and Wales and initially assessed at age 5 years. The cohort comprised 1242 monozygotic (56%) and 990 dizygotic (44%) twins; sex was evenly distributed within zygosity (1092 male [49%] and 1140 female [51%] children). Follow-up home visits were conducted when participants were aged 7 years (98% participation), 10 years (96%), 12 years (96%), and 18 years (93%). The cohort’s neighborhoods represented the full range of socioeconomic conditions in Great Britain. The participants’ addresses were a near-perfect match to the deciles of the UK government’s 2015 Lower-layer Super Output Area Index of Multiple Deprivation,^21^ which ranked neighborhoods by relative deprivation at an area level of approximately 1500 residents (eFigure in the Supplement). Approximately 10% of the E-Risk Study cohort filled each of the 10% bands of the Index of Multiple Deprivation, indicating that the cohort accurately represented the distribution of deprivation in Great Britain.

### Measures

#### Neighborhood Disadvantage or Ecological Risk Index

Neighborhood disadvantage was measured through an ecological risk assessment, which collected information from 4 independent sources of data (Box): (1) local government data, (2) criminal justice data, (3) systematic social observation (using Google Street View), and (4) surveys of neighborhood residents (conducted by the E-Risk Study team).

Box. **Four Data Sources for Assessing the Ecological Risk Index**

**Local Government Data**

Income and employment statistics
Health and disability records
Education, skills, and training attainment
Risk of crime
Barriers to housing and services
Quality of the local living environment

**Criminal Justice Data**

Total number of monthly street-level crimes

**Systematic Social Observation**

Environmental decay
Physical disorder
Perceived dangerousness

**Surveys of Neighborhood Residents**

Fear of crime
Direct victimization
Neighborhood problems
Social disconnectedness

We used these data sources to measure 4 neighborhood characteristics across childhood from age 5 to 17 years: deprivation, dilapidation, disconnection, and dangerousness. These measures have been previously described^22^ (eAppendix 2 in the Supplement).

For each of these 4 characteristics, we constructed a measure of ecological risk as follows. First, variables with skewed distributions were log transformed. Second, values were standardized to have a mean (SD) of 50 (10). Third, mean scores were calculated across measurement method within each domain. The resulting scales of deprivation, dilapidation, disconnection, and dangerousness were approximately normally distributed. Neighborhoods’ ecological risk levels on these 4 measures were correlated (Pearson *r* = 0.5-0.7) (eTable 2 in the Supplement). We computed the composite Ecological Risk Index by summing the values across the 4 measures. Ecological Risk Index values were generated for 2172 children (97% of the cohort).

#### Genome-Wide Quantification of DNA Methylation

The present epigenetic study used DNA from a single tissue: blood. Whole blood was collected in 10-mL K_2_ EDTA tubes from 1700 participants (82%) at age 18 years and was assayed for 1669 participants (31 blood samples were not useable because of low DNA concentration). DNA methylation was quantified using an Illumina 450k methylation assay (Infinium HumanMethylation450 BeadChip; Illumina, Inc) run on an array scanner (iScan System; Illumina, Inc). Blood samples from 1658 E-Risk Study participants passed the quality control pipeline (eAppendix 3 in the Supplement).

### Statistical Analysis 

**Figure 1.**
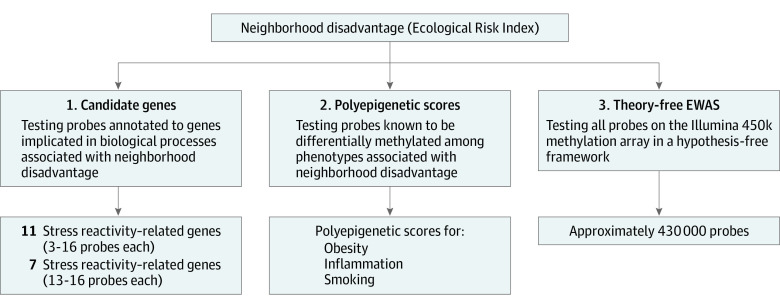
Analytic Approaches to Testing the Epigenetic Associations of Growing Up in Disadvantaged Neighborhoods The 3 separate preregistered approaches taken to test young-adult epigenetic associations with childhood neighborhood disadvantage. EWAS indicates epigenome-wide association study.

As mentioned earlier, we preregistered 3 approaches to studying the associations between neighborhood disadvantage and DNA methylation (Figure 1). These approaches involved testing probes annotated to candidate genes, using polyepigenetic scores that index phenotypes associated with growing up in disadvantaged environments, and conducting an EWAS.

#### Approach 1: Testing Candidate Genes

We interrogated 18 candidate genes that have been studied in the most detailed report about neighborhood disadvantage and DNA methylation.^18^ These genes included 7 stress reactivity–related (with 13-66 probes annotated to each) and 11 inflammation-related (with 3-16 probes annotated to each) genes (eTable 3 and eAppendix 4 in the Supplement).

We tested the associations between neighborhood disadvantage (assessed through the Ecological Risk Index) and probes annotated to these 18 candidate genes using generalized estimating equations following 3 steps. First, we applied a basic model in which probe methylation was regressed onto the neighborhood Ecological Risk Index and covariates of sex, methylation array control probe principal components indexing technical variation, and cell-type proportion estimates. A gene-wide significance threshold was derived for each gene by applying a Bonferroni correction to the nominal α = .05, with adjustment for number of probes tested (between 3 and 66) (eTable 3 in the Supplement). Second, we subjected the probes identified as gene-wide significant in the basic model to a smoking-adjusted model that controlled for a known implication of smoking for methylation data by adding information about the 18-year-old participants’ tobacco pack-years.^23^ Third, we subjected the probes identified as gene-wide significant in the smoking-adjusted model to a family socioeconomic status–adjusted model that added information about family social class (measured through a composite of parental income, education, and occupation).^24^

#### Approach 2: Testing Polyepigenetic Scores

Leveraging the observation that an EWAS of DNA methylation typically identifies multiple differently methylated CpG sites spread across multiple genes, we drew on previous EWAS reports about DNA methylation and obesity,^25^ inflammation,^26^ and tobacco smoking^27^ to create composite polyepigenetic scores that indexed the methylation correlates of these phenotypes. These phenotypes were chosen because they represented substantial public health and economic burden, were associated with neighborhood characteristics in previous studies,^28,29,30,31,32,33^ were prevalent among 18-year-old individuals in the UK at the time study data were collected, and had been subject to large-scale EWASs. Polyepigenetic scores were calculated by averaging the product of CpG probe intensities in the data and estimated coefficients across each of the CpG probes identified as epigenome-wide significant in previous meta-analyses of obesity,^25^ inflammation,^26^ and tobacco smoking.^27^ Scores were standardized to a mean (SD) of 0 (1) (eAppendix 5 in the Supplement).

We tested associations between neighborhood disadvantage and the polyepigenetic scores using ordinary least squares linear regression. Each score was examined using 3 models. First, we applied a basic model in which the polyepigenetic score was regressed onto the neighborhood Ecological Risk Index with the covariate of sex. Second, we applied a phenotype-adjusted model in which the polyepigenetic score was regressed onto neighborhood disadvantage and the covariates of sex and the age-18 phenotype relevant to the polyepigenetic score (obesity status, C-reactive protein level, and tobacco pack-years). This model was built to take into account the known implication of the phenotypes for the relevant polyepigenetic scores to ascertain whether the associations between the neighborhood and the epigenome were independent of individual health behaviors or conditions (eAppendix 5 in the Supplement). Third, we applied a family socioeconomic status–adjusted model in which the polyepigenetic score was regressed onto neighborhood disadvantage and the covariates of sex and family socioeconomic status.

#### Approach 3: Epigenome-Wide Association Study

In an EWAS, we tested the association between participants' childhood neighborhood disadvantage and their DNA methylation status across the epigenome (ie, on approximately 430 000 probes included in the data set from the Infinium HumanMethylation450 BeadChip array) using generalized estimating equations.

Three modeling steps were used. First, we applied a basic model in which probe methylation was regressed onto the neighborhood Ecological Risk Index and covariates of sex, methylation array control probe principal components indexing technical variation, and cell-type proportion estimates. An arraywide significance threshold of *P* < 1.16 × 10^−7^ was derived by applying a Bonferroni correction to the nominal α = .05, thereby adjusting for the 430 802 probes tested. Second, we subjected probes identified as arraywide significant in the basic model to a smoking-adjusted model that added information about 18-year-old participants’ pack-years to the basic model. Third, we subjected probes identified as arraywide significant in the smoking-adjusted model to a family socioeconomic status–adjusted model that added information about family social class.

#### Additional Statistical Notes

Because the E-Risk Study comprised twins, we accounted for the nonindependence of children within families in all models by adjusting the SEs, using the gee package for analyses conducted in R (R Foundation for Statistical Computing) and the Robust Cluster command for analyses conducted in Stata (StataCorp LLC). As a sensitivity test, all statistically significant models were subjected to additional statistical adjustment for twin zygosity status (monozygotic vs dizygotic), which did not change the results.

The premise and analysis plan for the present study were preregistered. Findings reported herein were checked for reproducibility by an independent data analyst, who recreated the code from the manuscript and applied it to a fresh data set.

Summary statistics of associations between neighborhood disadvantage and all DNA methylation probes on the methylation array are available on Open Science Framework.^34^ Methylation values were modeled as β values, which reflect the proportion of methylation, ranging from 0 to 1. Data analysis was performed from March 15, 2019, to June 30, 2019.

## Results

The Ecological Risk Index of childhood neighborhood disadvantage was generated for 2172 participants (97% of the full cohort [n = 2232]). Blood was collected from 1700 participants at age 18 years (82% of the cohort seen at that age [n = 2073]). Blood samples from 1658 participants passed the quality control pipeline (eAppendix 3 in the Supplement). Statistical analyses were performed on 1619 participants (73% of 2232), of whom 806 were female individuals (50%), with complete neighborhood and DNA methylation data (Table). No differences in socioeconomic background (*t*_2230_ = 1.174; *P* = .24) or neighborhood deprivation status, as measured by the Index of Multiple Deprivation (*t*_2154_ = –0.893; *P* = .37), were found between participants with or without complete neighborhood and methylation data.

**Table.** Demographic Characteristics of Environmental Risk Longitudinal Twin Study Participants

Variable	No. (%)			*P* value
	Full sample (n = 2232)	With complete data (n = 1619)	Without complete data (n = 574)	
Sex				
Female	1140 (51)	806 (50)	334 (55)	NA
Male	1092 (49)	813 (50)	279 (45)	NA
Zygosity				
Monozygotic	1242 (56)	916 (57)	326 (53)	NA
Dizygotic	990 (44)	703 (43)	287 (47)	NA
Family SES^a^				.24
Low	742 (33)	550 (34)	192 (31)	
Middle	738 (33)	532 (33)	206 (34)	
High	752 (34)	537 (33)	215 (35)	
Neighborhood deprivation status,^b^ mean (SD)	0.00 (1.00)	0.01 (1.00)	−0.03 (1.00)	.37
No.	2156	1564	592	NA

Abbreviations: NA, not applicable; SES, socioeconomic status.

^a^ Family SES was measured with a composite of parental income, educational level, and occupation divided into tertiles (ie, low [1], middle [2], and high-SES [3]).

^b^ Neighborhood deprivation status was measured with the UK government’s 2015 Lower-layer Super Output Area Index of Multiple Deprivation, which ranked British neighborhoods by relative deprivation at an area level of approximately 1500 residents; approximately 10% of the E-Risk Study cohort filled each of the index’s 10% bands. The deprivation measure was scaled within the full cohort to a mean (SD) of 1 (0).

### Neighborhood Disadvantage and Epigenetic Variation in Genes Involved in Inflammation and Stress Reactivity

Children raised in more socioeconomically disadvantaged neighborhoods did not display gene-wide significant differences in DNA methylation on most probes annotated to stress reactivity–related or inflammation-related genes. Overall, across the 317 probes annotated to the 18 candidate genes, associations crossed the threshold for gene-wide significance for only 1 probe that was annotated to the inflammation-related gene *NLRP12* (91662; cg07042144; β = 0.07; 95% CI, 0.03-0.11; *P* = .001). This association remained gene-wide significant (*P* < .006) after adjustment for participants’ tobacco pack-years (β = 0.06; 95% CI, 0.02-0.10; *P* = .003) but not after adjustment for family socioeconomic status (β = 0.06; 95% CI, 0.01-0.11; *P* = .02).

### Neighborhood Disadvantage and Polyepigenetic Scores Associated With Inflammation, Obesity, and Smoking Phenotypes

**Figure 2.**
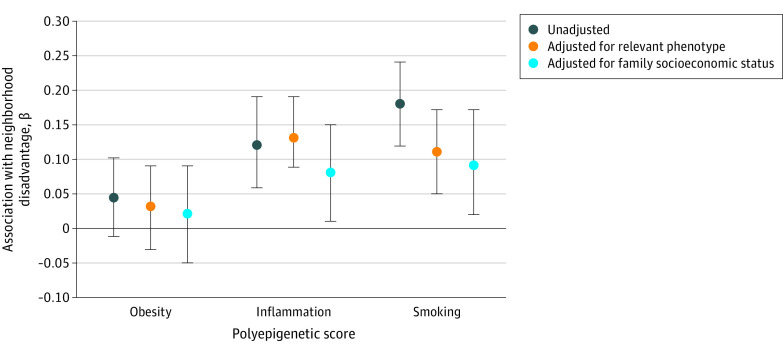
Association of Childhood Neighborhood Disadvantage With Young Adult Polyepigenetic Scores Error bars represent 95% CIs. Polyepigenetic scores indexed putative DNA methylation signatures for obesity, inflammation, and smoking, derived from meta-analyses of these phenotypes. All models were adjusted for sex. Additional covariates in the phenotype-adjusted model included obesity status, plasma C-reactive protein level, and pack-years for each of the relevant polyepigenetic scores. Family socioeconomic status was measured as a composite of parental income, educational level, and occupation.

We drew on published EWAS findings^25,26,27^ on 3 phenotypes of public health importance that were previously associated with neighborhood disadvantage (obesity, inflammation, and smoking). We constructed DNA methylation–based algorithms to capture manifold methylation differences in a single polyepigenetic score for each phenotype. Each resulting polyepigenetic score correlated statistically significantly with its phenotype at age 18 years in the E-Risk Study cohort (obesity: *r* = 0.35 [95% CI, 0.30-0.39; *P* < .001]; inflammation: *r* = 0.23 [95% CI, 0.18-0.28; *P* < .001]; and smoking: *r* = 0.45 [95% CI, 0.41-0.49; *P* < .001]). We then tested the associations between neighborhood disadvantage and these polyepigenetic scores (Figure 2). Three findings were notable.

First, children raised in more disadvantaged neighborhoods did not display statistically significantly greater obesity-related DNA methylation compared with their peers from less disadvantaged neighborhoods, as indexed by the obesity polyepigenetic score (β = 0.05; 95% CI, −0.01 to 0.11; *P* = .12). Second, children raised in more disadvantaged neighborhoods displayed greater inflammation-related DNA methylation compared with their peers from less disadvantaged neighborhoods, as indexed by the inflammation polyepigenetic score (β = 0.12; 95% CI, 0.06-0.19; *P* < .001). Adjusting for the relevant phenotype, C-reactive protein level did not alter the results (β = 0.13; 95% CI, 0.07-0.19; *P* < .001). Adjusting for family socioeconomic status attenuated the effect size to β = 0.07 (95% CI, 0.004-0.15), but the association remained statistically significant at *P* = .04. Third, children raised in more disadvantaged neighborhoods displayed greater smoking-related DNA methylation compared with their peers from less disadvantaged neighborhoods, as indexed by the smoking polyepigenetic score (β = 0.18; 95% CI, 0.11-0.25; *P* < .001). Adjusting for the relevant phenotype, tobacco pack-years attenuated the effect size to β = 0.11 (95% CI, 0.05-0.17), as did adjusting for family socioeconomic status (to β = 0.09; 95% CI, 0.02-0.17), although the association remained statistically significant in both cases (*P* < .05).

### Neighborhood Disadvantage and Epigenetic Variation Across the Entire Illumina 450K Array

**Figure 3.**
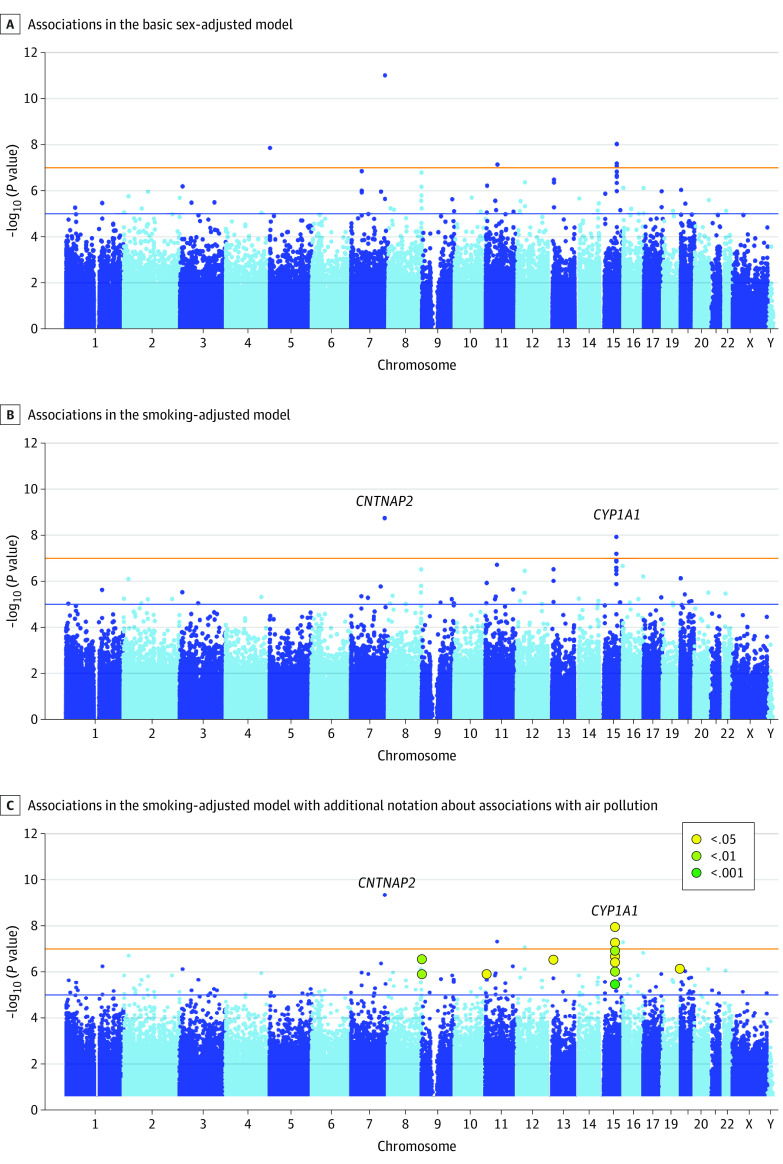
Association of Childhood Neighborhood Disadvantage With Epigenome-Wide DNA Methylation at Age 18 Years A, Associations with 6 probes passed the arraywide multiple testing threshold (*P* < 1.16 × 10^−7^; orange line), 3 of which were annotated to the *CYP1A1* gene on chromosome 15. Sixty-six probes passed the suggestive significance threshold (*P* < 1.0 × 10^−5^; blue line). B, Associations with 3 probes remained significant after adjustment for smoking status, 2 of which were annotated to the *CYP1A1* gene and 1 to the *CNTNAP2* gene. Two other probes annotated to the *CYP1A1* gene approached arraywide significance (*P* = 1.23 × 10^−7^ and *P* = 1.37 × 10^−7^) in the smoking-adjusted model. Fifty-nine probes passed the suggestive significance threshold, including 8 annotated to the *CYP1A1* gene. C, Smoking-adjusted associations shown with additional notation about probe associations with air pollution exposure. Large circles represent the top probes that were also significantly associated with nitrogen oxides (NOx) air pollution exposure, with darker color indicating smaller *P* values for the association. Of the top 20 probes from the smoking-adjusted epigenome-wide association study of neighborhood disadvantage, 12 were significantly associated with NOx air pollution exposure at the α = .05 level, 4 at the α = .01 level, and 1 at a level corrected for multiple testing of 20 tests (*P* < .001).

Children raised in more disadvantaged neighborhoods displayed arraywide significant differences (*P* < 1.16 × 10^−7^) in DNA methylation at age 18 years at 6 positions (Figure 3A) annotated to the *CNTNAP2* (26047), *CYP1A1* (1543), *AHRR* (57491), and *OR4C13* (283092) genes. Of these 6 arraywide significant probes, 3 were annotated to the *CYP1A1* gene. Probes annotated to the *CYP1A1* gene accounted for 8 of the top 20 most significant CpG sites, as ranked by *P* value (all *P* < 1.31 × 10^−6^). After adjustment for tobacco pack-years, 3 sites remained arraywide significant (Figure 3B), with 2 annotated to the *CYP1A1* gene (cg13570656 and cg00213123) and 1 annotated to the *CNTNAP2* gene (cg25949550). Two other *CYP1A1* gene sites approached the significance threshold, with cg17852385 reaching *P* = 1.23 × 10^−7^ and cg12101586 reaching *P* = 1.37 × 10^−7^. These 5 probes remained significant after adjustment for family socioeconomic status, although the effect sizes of the associations were attenuated (eTable 4 in the Supplement). Given that the *CNTNAP2* and *CYP1A1* genes were previously associated with maternal smoking while pregnant,^35^ we applied additional post hoc adjustment for maternal smoking to these 5 probes. The size of the associations with neighborhood disadvantage was attenuated, but all probes remained significant (eTable 4 in the Supplement).

### Air Pollution and Epigenetic Differences Associated With Neighborhood Disadvantage: Exploratory Secondary Analysis

Was air pollution implicated in young adult epigenetic differences associated with neighborhood disadvantage? The *CYP1A1* gene encodes a member of the cytochrome P450 superfamily of monooxygenase enzymes that is specifically involved in the metabolism of polycyclic aromatic hydrocarbons (PAHs),^36^ the toxic byproducts of organic material combustion found in cigarette smoke and emissions from residential heating, coke production, waste incineration, and internal combustion engines.^37^ After the primary EWAS finding of the associations between neighborhood disadvantage and multiple probes annotated to the *CYP1A1* gene that survived adjustment for study participants’ tobacco smoking and prenatal exposure to smoking, we designed post hoc exploratory follow-up analyses to test the hypothesis that toxic air pollutants other than cigarette smoke were associated with differential methylation in the *CYP1A1* gene across neighborhoods.

We used 2 measures of annual air pollution exposure estimated for the E-Risk Study participants at age 17 years: (1) exposure to nitrogen oxides (NOx), a regulated gaseous pollutant composed of nitrogen dioxide and nitric oxide and (2) exposure to fine particulate matter (PM_2.5_), a regulated aerosol pollutant with suspended solid and liquid particles smaller than 2.5 μm in diameter. Hourly pollution exposure estimates were modeled down to individual streets on which participants lived and spent most of their time and were calculated to estimate the mean pollutant-level exposure across 1 year (2012) preceding the assessment of participants at age 18 years (eAppendix 6 in the Supplement). Although not direct measures of PAH, NOx and PM_2.5_ represent byproducts of the incomplete combustion of organic material, with NOx, in particular, associated with common PAH sources.^37^ In general, participant exposure to NOx (mean [SD] annual level, 25.71 [16.28] μg/m^3^) declined within World Health Organization guidelines for nitrogen dioxide (40 μg/m^3^), a component of NOx, whereas exposure to PM_2.5_ (mean [SD] annual level, 11.24 [2.18] μg/m^3^) exceeded World Health Organization guidelines (10 μg/m^3^).^38^ Levels of both pollutants were higher in more disadvantaged neighborhoods (*r* = 0.32 [*P* < .001] between neighborhood disadvantage and exposure to NOx and *r* = 0.22 [*P* < .001] between neighborhood disadvantage and exposure to PM_2.5_).

Using ordinary least squares multiple regression, we tested the association of the top 20 differentially methylated probes identified in the smoking-adjusted EWAS of neighborhood disadvantage, as ranked by *P* value, with estimates of participant exposure to NOx and PM_2.5_ (eTable 5 in the Supplement). With NOx, associations for 12 of the top 20 probes achieved significance at the α = .05 level, 4 probes at the α = .01 level, and 1 probe at a level corrected for multiple testing of 20 tests, *P* < .001 (Figure 3C). With PM_2.5_, associations for 3 of the top 20 probes achieved significance at the α = .05 level, 2 probes at the α = .01 level, and 0 probe at a level adjusted for 20 tests, *P* < .001.

## Discussion

Three findings emerged from this longitudinal cohort study of the association between childhood neighborhood socioeconomic disadvantage and young adult DNA methylation. First, children raised in more disadvantaged neighborhoods did not, when compared with their peers who were raised in less disadvantaged neighborhoods, display any marked pattern of differential DNA methylation among probes indexed to candidate genes that were tested in previous epigenetic research on neighborhood effects. This finding represents a failure to replicate in a young adult sample a previous report about DNA methylation among older adults aged 45 to 84 years living in disadvantaged neighborhoods.^18^ This inability to replicate the result may reflect differences in accumulated epigenetic burden between those who have lived in disadvantaged neighborhoods for a short time (≤18 years) and those who have lived there for multiple decades. It likely does not result from differences in power, as this study had a larger sample than the work by Smith et al.^18^

Second, children raised in more disadvantaged neighborhoods displayed greater DNA methylation associated with inflammation and tobacco smoking but not with obesity. This finding represents a partial replication of a previous report.^18^ These results held even after adjustment for inflammation and smoking phenotypes. Epigenetic signatures of inflammation and smoking without elevated C-reactive protein levels and smoking behavior may be explained by 3 hypotheses: (1) they could represent the historical trace of a former phenotype that is no longer present; (2) they could signal a future condition that is yet to emerge, to the extent that these epigenetic signatures are not outcomes but causes; and (3) they could indicate the presence of phenotypes associated with inflammation and smoking that were not observed in this study, such as non–C-reactive protein–related inflammation and non–tobacco smoke–related air pollutant exposure. We were unable to empirically adjudicate between these 3 possibilities.

Third, in a hypothesis-free EWAS, 18-year-old participants raised in more disadvantaged neighborhoods displayed differential methylation of probes annotated to the *CNTNAP2* and *CYP1A1* genes. Adjustment for tobacco smoking, family socioeconomic status, and in utero exposure to maternal smoking reduced the size of these associations but did not account for them entirely. The *CYP1A1* gene is putatively involved in the metabolism of PAH found in cigarette smoke and ambient outdoor air pollution. Exploratory follow-up tests using 2 measures of air pollutant exposure (NOx and PM_2.5_) identified statistically significant associations between neighborhood disadvantage–related probes, particularly at the *CYP1A1* gene, and adolescent exposure to air pollution. Air pollution may be associated with epigenetic differences among young adults raised in different neighborhoods. Notably, the *CYP1A1* gene is believed to encode an enzyme specifically involved in the activation of PAH carcinogenic intermediates^36,39^; the gene’s activity has consequently been associated with lung cancer risk after PAH exposure.^39,40,41^ Evidence suggests that the EWAS-identified *CYP1A1* probes are located within a *CYP1A1* gene-enhancer region.^42^ Thus, differential expression of the *CYP1A1* gene may represent a pathway linking the childhood neighborhood environment to risk of disease in adulthood.

To our knowledge, this cohort study is the largest and most comprehensive test of the hypothesis that epigenetic regulation may be 1 biological pathway through which neighborhood disadvantage gets under the skin to engender long-term health disparities. If confirmed, these findings suggest that policy interventions at the neighborhood level could alter long-term child health trajectories.

### Limitations

This study has some limitations. First, we used DNA only from blood. The findings may not generalize to other tissues. Second, across all probes on the array, the effect sizes were small. In the top 20 EWAS-identified probes, study participants raised in the least disadvantaged neighborhoods (bottom 10% on the Ecological Risk Index) displayed, in general, between 1% and 4% difference in DNA methylation compared with participants raised in the most disadvantaged neighborhoods (top 10% on the Ecological Risk Index). Differences of this size may not have practical biological effects, although small shifts in methylation can have meaningful implications at the cell level.^43^ Third, although the availability of air pollution exposure data allowed for exploratory follow-up tests, no direct measure of PAH exposure or air pollution exposure across childhood was available. The air pollution findings should be considered suggestive. Fourth, this study involved only 1 cohort in only 1 country. To encourage replication in other settings, particularly among other long-term studies of children and adolescents, we have made the results of this study available on Open Science Framework,^34^ and we encourage replication. Fifth, this study was observational and did not establish causation.

## Conclusions

This study presents evidence that neighborhood disadvantage is associated with DNA methylation differences in genes involved in inflammation, exposure to tobacco smoke, and metabolism of toxic air pollutants. Collectively, these results suggest that children raised in disadvantaged neighborhoods enter adulthood epigenetically distinct from their more advantaged peers.

## 

## Notes

Supplement. **eTable 1.** Previous Studies Characterizing the Association Between Aspects of the Neighborhood Environment and the Epigenome **eAppendix 1.** Additional Details on the E-Risk Study Sample **eFigure.** The E-Risk Study Families’ Addresses Are a Near-Perfect Match to the Deciles of the UK Government’s Index of Multiple Deprivation **eAppendix 2.** Additional Details on the Neighborhood Characteristics and Data Sources for the Neighborhood Disadvantage or Ecological Risk Index **eTable 2.** Means and Standard Deviations of Neighborhood Measures and Their Correlations With One Another **eAppendix 3.** Additional Details on the Measurement of DNA Methylation **eTable 3.** Replication Candidate Stress Reactivity-Related and Inflammation-Related Genes **eAppendix 4.** Additional Details on the Selection of Probes for the Candidate Genes Analysis **eAppendix 5.** Additional Details on the Creation of Polyepigenetic Scores (for Obesity, Inflammation, and Smoking) and the Measurement of Related Phenotypes **eTable 4.** Association Between Neighborhood Disadvantage and DNA Methylation **eAppendix 6.** Additional Details on the Measurement of Air Pollution Exposure **eTable 5.** Association of the Top 20 Neighborhood Disadvantage EWAS Probes With Estimates of Participants’ Exposure to NOx and PM2.5 **eReferences**
