# Supplementary material for: Association of Neighborhood Disadvantage in Childhood With DNA Methylation in Young Adulthood
Source: JAMA Netw Open. 2020 Jun 1;3(6):e206095. doi: 10.1001/jamanetworkopen.2020.6095 (PMC7265095; doi:10.1001/jamanetworkopen.2020.6095)
Supplement: Supplement. — eTable 1. Previous Studies Characterizing the Association Between Aspects of the Neighborhood Environment and the Epigenome eAppendix 1. Additional Details on the E-Risk Study Sample eFigure. The E-Risk Study Families’ Addresses Are a Near-Perfect Match to the Deciles of the UK Government’s Index of Multiple Deprivation eAppendix 2. Additional Details on the Neighborhood Characteristics and Data Sources for the Neighborhood Disadvantage or Ecological Risk Index eTable 2. Means and Standard Deviations of Neighborhood Measures and Their Correlations With One Another eAppendix 3. Additional Details on the Measurement of DNA Methylation eTable 3. Replication Candidate Stress Reactivity-Related and Inflammation-Related Genes eAppendix 4. Additional Details on the Selection of Probes for the Candidate Genes Analysis eAppendix 5. Additional Details on the Creation of Polyepigenetic Scores (for Obesity, Inflammation, and Smoking) and the Measurement of Related Phenotypes eTable 4. Association Between Neighborhood Disadvantage and DNA Methylation eAppendix 6. Additional Details on the Measurement of Air Pollution Exposure eTable 5. Association of the Top 20 Neighborhood Disadvantage EWAS Probes With Estimates of Participants’ Exposure to NOx and PM2.5 eReferences [file jamanetwopen-3-e206095-s001.pdf]

Supplementary Online Content

Reuben A, Sugden K, Arseneault L, et al. Association of neighborhood disadvantage in childhood with DNA methylation in young adulthood. *JAMA Netw Open.* 2020;3(6):e206095. doi:10.1001/jamanetworkopen.2020.6095

eTable 1. Previous Studies Characterizing the Association Between Aspects of the Neighborhood Environment and the Epigenome

eAppendix 1. Additional Details on the E-Risk Study Sample

eFigure. The E-Risk Study Families' Addresses Are a Near-Perfect Match to the Deciles of the UK Government's Index of Multiple Deprivation

eAppendix 2. Additional Details on the Neighborhood Characteristics and Data Sources for the Neighborhood Disadvantage or Ecological Risk Index

eTable 2. Means and Standard Deviations of Neighborhood Measures and Their Correlations With One Another

eAppendix 3. Additional Details on the Measurement of DNA Methylation

eTable 3. Replication Candidate Stress Reactivity-Related and Inflammation-Related Genes

eAppendix 4. Additional Details on the Selection of Probes for the Candidate Genes Analysis

eAppendix 5. Additional Details on the Creation of Polyepigenetic Scores (for Obesity, Inflammation, and Smoking) and the Measurement of Related Phenotypes

eTable 4. Association Between Neighborhood Disadvantage and DNA Methylation

eAppendix 6. Additional Details on the Measurement of Air Pollution Exposure

eTable 5. Association of the Top 20 Neighborhood Disadvantage EWAS Probes With Estimates of Participants' Exposure to NOx and PM2.5

eReferences

This supplementary material has been provided by the authors to give readers additional information about their work.

eTable 1. Previous Studies Characterizing the Association Between Aspects of the Neighborhood Environment and the Epigenome

First author (year)	Sample/setting	Neighborhood environment measures	Methylation target(s)	Methylation analysis	Source / tissue	Results
Coker et al.[1]	N = 241 maternal/infant pairs who participated in the Center for Health Assessment of Mothers and Children of Salinas (CHAMACOS) (Salinas, CA, USA).<br>**Age:** 25.8 ± 5.3 years<br>**Ethnicity:**<br>Latina: 232 (96.2%)<br>White: 5 (2.1%)<br>Other: 4 (1.7%)	2000 U.S. Census Bureau census tract for:<br>• Percentage of homes below poverty line;<br>• Median household income; and<br>• Rates of high school completion.	LINE-1 and Alu (proxy measures of global DNA methylation)	Bisulfite pyro-sequencing	Umbilical cord blood	Living in the highest poverty neighborhood quartile was associated with higher cord blood LINE-1 methylation compared with living in the lowest poverty neighborhood quartile after adjustment for maternal smoking during pregnancy, maternal age, diet quality during pregnancy, years living in the USA, and prenatal urinary monobenzyl phthalate (MBzP) exposure ($\beta = 0.78$; 95% Cl 0.06, 1.50; $p = 0.03$).<br>No significant differences in LINE-1 methylation by neighborhood income levels or by neighborhood educational attainment in the crude or adjusted models.<br>No significant associations of neighborhood measures with Alu methylation.

©2020 Reuben A et al. *JAMA Network Open*

Janusek et al.[2]	N = 34 emerging adult males (Chicago, IL, USA).<br>**Age:** 25.4 ± 2.3 years<br>**Ethnicity:**<br>African American: 34 (100%)	Self-report on the Survey of Exposure to Community Violence (SECV) for direct victimization by violence / witnessing of violence on another.	IL-6 promoter region (inflammatio-n)	Bisulfite pyro-sequencing	Peripheral blood mononucl-ear cells	Indirect exposure to neighborhood violence was not related to DNA methylation.
King et al.[3]	N = 489 mothers from the Newborn Epigenetic Study (NEST) (Durham, NC, USA)<br>**Ethnicity:**<br>White: 240 (49.1)<br>Black: 193 (39.5)<br>Asian: 10 (2.0)<br>Other: 36 (7.4)	2010 U.S. Census Bureau census tract for economic indicators such as:<br>• Percent families with income below the poverty level;<br>• Percent of households on public assistance;<br>• Percent of households with an unmarried female head;<br>• Percent of the civilian labor force over age 16 unemployed.	MEG3 (tumor suppressor)	Bisulfite pyro-sequencing	Umbilical cord blood leukocytes	Neighborhood disadvantage was associated with significantly higher MEG3 methylation after controlling for maternal race/ethnicity, gender of newborn, mother's years of education, maternal household income, pre-pregnancy body-mass index, cigarette smoking, and use of antibiotics during pregnancy ($\beta = 0.76$, $p = 0.002$). A one standard deviation increase in prenatal neighborhood disadvantage also predicted higher MEG3 methylation ($p < 0.05$).

Lei et al.[4]	N = 99 women randomly sampled from the Family and Community Health Study (FACHS)<br>**Age:** 48.33 ± 9.30 years<br>**Ethnicity:**<br>African American: 99 (100%)	A summed score of neighborhood crime via two self-report measures:<br>• 11-item scale on the extent to which various criminal acts were a problem in the neighborhood (e.g., drinking in public); and<br>• 6-item neighborhood deviance scale assessing whether residents perceive their neighborhoods as high in crime.	5-HTT (serotonin transporter) 7 CpG sites within the CpG island associated with 5-HTT	Illumina Infinum 450K Human Methylation Beadchip Illumina HumanHT-12 v4 Expression BeadChip to measure gene expression.	Peripheral blood	No differences in neighborhood crime between methylation of 5-HTTLPR.<br>Interaction of 5-HTTLPR with neighborhood crime on depressive symptoms was mediated by5-HTT methylation (indirect effect = 0.062, $p < 0.031$).<br>There was no indirect effect of neighborhood crime on depressive symptoms through 5-HTT methylation when the respondents carried long 5-HTTLPR allele. However, there was an indirect effect of neighborhood crime on depressive symptoms though 5-HTT methylation when the respondents carried a short 5-HTTLPR allele (indirect effect = 0.064, $p = 0.015$).
Lei et al.[5]	N = 100 women randomly sampled from the Family and Community Health Study (FACHS)<br>**Age:** 48.52 ± 9.30 years<br>**Ethnicity:**<br>African American: 100	2010 U.S. Census Bureau census tract information for economic indicators including:<br>• Median household	Methylomic age based on weighted sum of methylation values at 71 CpG sites associated	Illumina Infinum 450K Human Methylation Beadchip.	Peripheral blood	Neighborhood disadvantage was associated with greater methylomic age. 1 SD increase in neighborhood disadvantage was associated with a methylomic profile akin to 9 months of additional biological aging.

	(100%)	income,<br>• Percent unemployed males,<br>• Percent below the poverty threshold,<br>• Percent who are single-mother families, and<br>• Percent receiving public assistance.	with biological aging.			
Smith et al.[6]	N = 1226 random subsample of the longitudinal, population-based Multi-ethnic Study of Atherosclerosis (MESA) (USA).<br>**Age in years:** N (%)<br>55–65: 463 (38%)<br>66–75: 397 (33%)<br>76–85: 300 (24%)<br>85–95: 66 (5%)<br>**Ethnicity:**<br>White: 581 (47%)<br>African American: 263	Neighborhood-level socioeconomic disadvantage index created from 2000 U.S. Census tract level variables (e.g., education, income, wealth, poverty, employment, housing); and<br><br>Neighborhood social environment index created from self-report questionnaire with scales on esthetic quality	18 candidate genes related to stress reactivity (AVP, BDNF, CRF, FKBP5, GR, OXTR, SLC6A4) and inflammation (CD1D, CCL1, F8, IL8, KLRG1, LTA4H, NLRP12, PYDC1, SLAMF7,	Illumina Infinum 450K Human Methylation Beadchip Illumina HumanHT-12 v4 Expression BeadChip to measure gene expression.	Monocyte-s from peripheral blood sample; morning draw after 12 h fast.	Neighborhood socioeconomic disadvantage was related to DNA methylation in 2 stress-related genes (CRF, SLC6A4) and 2 inflammation-related genes (F8, TLR1) after adjustment for age, gender, race/ethnicity, childhood SES, adult SES, and enrichment scores for each of the 4 major blood cell types (neutrophils, B cells, Tcells, and natural killer cells). However, the results varied across genes and site types (promoter vs. shore/shelf).<br>Neighborhood socioeconomic disadvantage was associated with increased methylation in non-promoter sites of CRF, F8, and TLR1; shore/shelf sites of AVP and SLC6A4; and non-shore sites of F8.<br>Neighborhood socioeconomic disadvantage

	(22%)<br>Hispanic: 382 (31%)	(e.g., lots of trash and litter on the street), safety (e.g., violence is a problem in my neighborhood), and social cohesion (e.g., people in my neighborhood can be trusted).	TLR1, TLR3)			was associated with decreased methylation in non-shore/shelf sites of CRF (all FDR q-values ranging from 0.01–0.10).<br>Neighborhood social environment was associated with methylation in 4 stress-related genes (AVP, BDNF, AKBP5, SLC6A4) and 7 inflammation-related genes (CCL1, CD1D, F8, KLRG1, NLRP12, SLAMF7, TLR1) after adjustment for age, gender, race/ethnicity, childhood SES, adult SES and enrichment scores for each of the 4 major blood cell types (neutrophils, B cells, Tcells, and natural killer cells). Similarly, the results were dependent upon the genes and site type. Neighborhood social environment was associated with increased methylation in promoter sites of AVP, CCL1, CD1D, F8, KLRG1, and SLAMF7; non-promoter sites of AVP, BDNF, NLRP12, and TLR1; shore/shelf sites of AVP, FKBP5; SLC6A4; and non-shore/shelf sites of CD1D, F8, SLAMF7, and TLR1.<br>Neighborhood social environment was associated with decreased methylation in non-promoter sites of CCL1 and KLRG1; shore/shelf sites of F8; and non-shore/shelf sites of FKBP5 (all FDR q-values ≤ 0.10).
Wriggles-worth et al.[7]	33 members of the Orygen Adolescent Development Study<br>**Age:** 12.8 ± 0.3 years	Australian 2006 Socio-Economic Indexes for Areas (SEIFA) using	BDNF IV candidate gene	Bisulfite pyro-sequencing	Buccal swab	Neighborhood disadvantaged was negatively associated with BDNF methylation on the exon IV promoter site.

	**Ethnicity:** Not reported	consensus data on financial burden, crime rates, and health outcomes in a geographical area of approximately 250 homes.				BDNF exon IV promoter site methylation mediated associations found between neighborhood disadvantage and measures of brain cortical thickness, namely thickness of the lateral orbitofrontal cortex.

Adapted from Giurgescu et al.[8]

©2020 Reuben A et al. *JAMA Network Open*

eAppendix 1. Additional Details on the E-Risk Study Sample

Participants are members of the Environmental-Risk (E-Risk) Longitudinal Twin Study, a nationally representative sample of children born in 1994 and 1995 in England and Wales (N=2232). Briefly, the E-Risk sample was constructed in 1999–2000, when 1116 families with same-sex 5-year-old twins (93% of those eligible) participated in home-visit assessments. The full sample comprised 56% monozygotic (MZ) and 44% dizygotic (DZ) twin pairs; sex was evenly distributed within zygosity (49% male). Families were recruited to represent the UK population of families with newborns in the 1990s, based on residential location throughout England and Wales and mothers' age (teenaged mothers with twins were over-selected to replace high-risk families who were selectively lost to the register through non-response. Older mothers having twins via assisted reproduction were under-selected to avoid an excess of well-educated older mothers). The cohort's neighborhoods represent the full range of socioeconomic conditions in Great Britain, as reflected in the families' distribution on a neighborhood-level socioeconomic index (ACORN [A Classification of Residential Neighborhoods], developed by CACI Inc. for commercial use).[9] E-Risk families' ACORN distribution closely matches that of households nation-wide: 25.6% of E-Risk families live in "wealthy achiever" neighborhoods compared to 25.3% nationwide; 5.3% vs. 11.6% live in "urban prosperity" neighborhoods; 29.6% vs. 26.9% live in "comfortably off" neighborhoods; 13.4% vs. 13.9% live in "moderate means" neighborhoods; and 26.1% vs. 20.7% live in "hard-pressed" neighborhoods. E-Risk underrepresents "urban prosperity" neighborhoods because such households are likely to be childless. **Figure S1** shows E-Risk families' addresses are a near-perfect match to the deciles of the UK

©2020 Reuben A et al. *JAMA Network Open*

government's 2015 Lower-layer Super Output Area (LSOA) Index of Multiple Deprivation, which ranks British neighborhoods in terms of relative deprivation at an area level of approximately 1,500 residents; approximately 10% of the E-Risk cohort fills each of the Index's 10% bands, indicating that the E-Risk cohort accurately represents the distribution of deprivation in the UK.

Follow-up home visits were conducted when the participants were aged 7 (98% participation), 10 (96%), 12 (96%), and, most recently, 18 (93%) years. The Joint South London and Maudsley and the Institute of Psychiatry Research Ethics Committee approved each phase of the study. Parents gave informed consent and twins gave assent between 5–12 years and then informed consent at age 18. Further details about the sample are reported elsewhere.[10]

eFigure. The E-Risk Study Families' Addresses Are a Near-Perfect Match to the Deciles of the UK Government's Index of Multiple Deprivation

The histogram shows E-Risk families' addresses are a near-perfect match to the deciles of the UK's 2015 Lower-layer Super Output Area (LSOA) Index of Multiple Deprivation (IMD) which averages 1,500 residents; approximately 10% of the E-Risk cohort (dotted red line) fills each of the IMD's 10% bands, indicating that the E-Risk cohort accurately represents the distribution of deprivation in the UK.

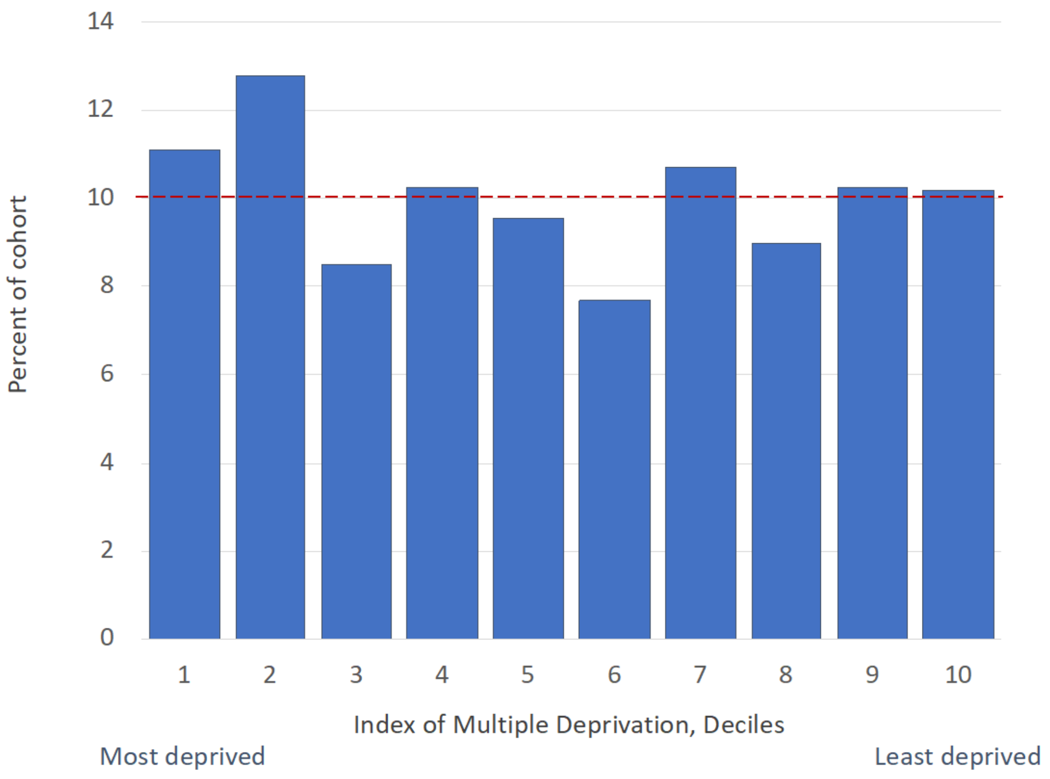

Notes. The UK Ministry of Housing, Communities & Local Government Index of Multiple Deprivation is an official measure of relative deprivation for every LSOA small area (approximately 1,500 residents or 650 households each) in England.

©2020 Reuben A et al. *JAMA Network Open*

eAppendix 2. Additional Details on the Neighborhood Characteristics and Data Sources for the Neighborhood Disadvantage or Ecological Risk Index

Neighborhood disadvantage was measured through ecological risk assessment conducted by combining information from four independent sources of data: 1) geodemographic data from local governments; 2) official crime data from the UK Police; 3) Google Street View-based Systematic Social Observation (SSO);[11] and, 4) surveys of neighborhood residents having the same postcode as each Study family, conducted by the E-Risk Study team. We used these data sources to measure four neighborhood characteristics across childhood (from ages 5 to 17 years): deprivation, dilapidation, disconnection, and dangerousness.

Neighborhood characteristics measured

Deprivation was measured with the UK Ministry of Housing, Communities & Local Government Index of Multiple Deprivation, an official measure of relative deprivation for every LSOA small area (approximately 1,500 residents or 650 households each) in England.

Dilapidation was measured from resident ratings of problems in their neighborhood (e.g. litter, vandalized public spaces, vacant storefronts) and independent raters' assessments of these same problems based on the "virtual walk-through" using Google Street View.

Disconnection was measured from resident ratings assessing neighborhood collective efficacy and social connectedness. *Neighborhood collective efficacy* was

assessed via the resident survey using a previously validated 10-item measure of social control and social cohesion [12]. Residents were asked about the likelihood that their neighbors could be counted on to intervene in various ways if, for example: "children were skipping school and hanging out on a street corner," "children were spray-painting graffiti on a local building." They were also asked how strongly they agreed that, for example: "people around here are willing to help their neighbors," "this is a close-knit neighborhood" (item responses: 0-4). *Social connectedness* was assessed based on indicators of *intergenerational closure* ("If any of your neighbors' children did anything that upset you would you feel that you could speak to their parents about it?"), *reciprocated exchange* (e.g., "Would you be happy to leave your keys with a neighbor if you went away on holiday?") and *friendship ties* (e.g., "Do you have any close friends that live in your neighborhood") among neighbors [13].

Dangerousness was measured from police records of crime incidence, from neighborhood residents' ratings of how much they feared for their safety and whether they had been victimized, and from independent raters' assessments of neighborhood safety based on the "virtual walk-through" using Google Street View.

Data sources used

1. Geodemographic Data from Local Governments. We obtained information about the Index of Multiple Deprivation from the Department for Communities and Local Government. The Index is the official measure of relative deprivation for neighborhoods in England. Every small area in England is ranked from 1 (most deprived area) to 32,844 (least deprived area), these rankings are then converted into deciles. The Index of Multiple Deprivation is created based on 37 separate indicators, that are organized in seven domains of deprivation (Income Deprivation,

Employment Deprivation; Education, Skills and Training Deprivation; Health Deprivation and Disability; Crime; Barriers to Housing and Services; and Living Environment Deprivation), and combined with appropriate weights to calculate the Index of Multiple Deprivation (IMD). Households were assigned a neighborhood IMD based on street address at the time of the age-5, age-7, age-10, and age-12 in-home visits using scores from the 2015 IMD. We analyzed the average IMD value across these four measurements.

2. Official Crime Data. We measured local area crime by mapping a 1-mile radius around each E-Risk Study family's home and tallying the total number of crimes that occurred in the area each month in 2011, when the participants were age 17. Street-level crime data, including information on the type of crime, date of occurrence, and approximate location, were accessed online as part of an open data sharing effort about crime and policing in England and Wales (https://data.police.uk/) and geocoded to the home address of the Study members. An Application Program Interface was used to extract street-level crime data for each of the geospatial coordinates marking the family's home. For a full description see: https://data.police.uk/about/#location-anonymisation.

3. Google Street View Virtual Systematic Social Observation (SSO). The Google Street View SSO consisted of trained raters taking a "virtual walk" through the neighborhoods of the E-Risk families based on street address at the time of the age-12 home visit. Raters then coded neighborhoods based on what they saw on that virtual walk. Street View is a freely available tool that generates panoramic street-level views using high definition images taken from camera-equipped cars. Signals from global positioning devices are used to accurately position images in the online maps. To avoid gaps in the imagery, adjacent cameras on the car take overlapping pictures and the images are then stitched together to create a continuous 360-degree image of the street. Images are then smoothed and re-projected onto a sphere to create the image displayed in Street View (see Main Article **Figure 1**). To protect the privacy of individuals, face- and license-blurring technology is applied to ensure that people on

©2020 Reuben A et al. *JAMA Network Open*

the street and cars in the photographs cannot be identified. Google Street View came online in the United Kingdom in March 2009 and by March 2010, 94% of the E-risk children's neighborhoods were available for viewing. The Google Street View SSO was completed by adapting SSO instruments for the virtual context and training raters to reliably code neighborhood features while taking a virtual walk down the street. We have reported full details of the Google Street View SSO method, inter-rater reliability and predictive validity of the measures elsewhere.[11] We analyzed Google Street View SSO measures of environmental decay and disorder and perceived dangerousness.

4. Resident Surveys. A survey of residents living alongside E-Risk families was conducted when the children were 13-14 years of age to capture neighborhood-level social processes that cannot easily be captured via official records or direct observation. The sampling frame for the Neighborhood Survey was drawn using UK-Info Pro V13 http://www.192.com/products/. The survey responses were anonymous; no identifying information was collected. In Britain, a postcode area typically contains 15 households, with at most 100 households (e.g., a large apartment block). Therefore, survey respondents were typically living on the same street or within the same apartment block as the children in our study. Surveys were mailed to every household in the postcode registered to the electoral role, with the exception of the E-Risk family, resulting in 20,529 surveys being mailed to households to capture information on E-Risk families. On average, we received 5 (SD=3) completed surveys per neighborhood (range=0-18 respondents). We achieved at least 3 responses for 80% of target neighborhood and at least 2 responses for 95% (resulting in a total of 5601 completed questionnaires). Survey responses were received for N=1,077 of the 1,116 families in the study. We analyzed survey measures of the following neighborhood-level social processes: fear of crime, direct victimization, neighborhood problems, and social disconnectedness.

eTable 2. Means and Standard Deviations of Neighborhood Measures and Their Correlations With One Another

					Pearson Correlation			
		M	SD		(1)	(2)	(3)	(4)
(1)	**Composite Ecological Risk Index**	200.06	33.33		---			
(2)	Deprived	50	10		.80	---		
(3)	Dilapidated	50	10		.84	.54	---	
(4)	Disconnected	50	10		.80	.48	.55	---
(5)	Dangerous	50	10		.90	.65	.70	.63

Notes. All correlations are significant, p<.001

©2020 Reuben A et al. *JAMA Network Open*

eAppendix 3. Additional Details on the Measurement of DNA Methylation

Our epigenetic study used DNA from a single tissue: blood. At age 18, whole blood was collected from 82% (N=1700) of the participants in 10mL K_2EDTA tubes. DNA was extracted from the buffy coat using a Flexigene DNA extraction kit (Qiagen, Hilden, Germany) following manufacturer's instructions. Study members who did not provide blood provided buccal swabs, but these were not included in our methylation analysis to avoid tissue-source confounds. These data have been previously described[14] and are accessible from the Gene Expression Omnibus (accession code: GSE105018).

We assayed 1669 blood samples (out of 1700); 31 samples were not useable (e.g., due to low DNA concentration). ~500ng of DNA from each sample was treated with sodium bisulfite using the EZ-96 DNA Methylation kit (Zymo Research, CA, USA). DNA methylation was quantified using the Illumina Infinium HumanMethylation450 BeadChip ("Illumina 450K array") run on an Illumina iScan System (Illumina, CA, USA). Twin pairs were randomly assigned to bisulfite-conversion plates and Illumina 450K arrays, with siblings processed in adjacent positions to minimize batch effects. Fully methylated control samples (CpG Methylated HeLa Genomic DNA; New England BioLabs, MA, USA) were included in a random position on each plate; the distinct DNA methylation profile of this sample enabled us to confirm the experiment was successful and to ensure there were no plate mix-ups or rotations.

Data were imported using the methylumIDAT function in 'methylumi,' and subjected to quality control analyses. First, we excluded all samples with median methylated ('M') and unmethylated ('U') intensities <2500. Second, using the ten control

probes included on the 450K array, we examined the efficiency of the sodium bisulfite conversion reaction; samples were excluded if their "conversion score" was <80. Third, multidimensional scaling was performed for DNA methylation probes on each of the sex chromosomes and compared to the reported gender. Fourth, to confirm genetic identity of the DNA samples, we assessed genotype concordance between SNP probes on the 450K array and data generated using Illumina OmniExpress24v1.1 genotyping BeadChips.

Data were processed in R with the pfilter function from the 'wateRmelon' R package[15] excluding 0 samples with >1% of sites with a detection p value >0.05, 567 sites with beadcount <3 in 5% of samples and 1448 probes with >1% of samples with detection p value >0.05. The data were normalized with the dasen function from the 'wateRmelon' R package.[15] Prior to any analyses, probes with common (> 5% MAF) SNPs within 10 bp of the single base extension and probes with sequences previously identified as potentially hybridizing to multiple genomic loci were excluded,[16,17] resulting in a final dataset of 430,802 probes. Samples from 1,658 E-Risk participants passed our QC pipeline.

Statistical analyses were performed on n=1619 with complete neighborhood and DNA methylation data. There were no differences in socioeconomic background (t(2230)=1.174, p=.241) or neighborhood deprivation status (measured by the UK government Index of Multiple Deprivation) (t(2154)=-0.893, p=.372) between participants with and without complete neighborhood and DNA methylation data.

To adjust for cellular heterogeneity that can affect methylation-based scores, all analyses testing associations with methylation beta values included a cell-type distribution variable. Cell-type composition was estimated using the reference-based

method developed by Houseman and colleagues[18] and Horvath.[19] Using this approach, we estimated cell-type proportions in whole blood for CD4+ T cells, Natural Killer cells, granulocytes, monocytes, naïve CD8 T cells, CD8+CD28-CD45RA- T cells and PlasmaBlasts.

Power sensitivity analysis of the analytic sample (n=1619) conducted using the G*Power package (v3.1) revealed sensitivity to detect effect sizes as low as Cohen's f^2 = 0.008 at power = 0.95 in the most saturated models. This is considered a small effect, accounting for <1% of the variance in the outcome variable (given by the equation: proportion of variance = $\frac{f^2}{(1+f^2)}$).[20]

eTable 3. Replication Candidate Stress Reactivity-Related and Inflammation-Related Genes

Domain	Gene	# of Probes	Codes for
Involved in stress response / stress reactivity	NR3C1	42	Glucocorticoid receptor
	FKBP5	33	Regulator of the glucocorticoid receptor network
	BDNF	66	Brain-derived neurotrophic factor
	AVP	17	Neuropeptide vasopressin
	CRHR1	31	Corticotropin-releasing hormone receptor
	SLC6A4	16	Serotonin transporter gene
	OXTR	13	Oxytocin receptor
Involved in immune response / inflammation	CD1D	16	Glycoprotein lipid antigen CD1
	CCL1	8	Glycoprotein inflammatory cytokine
	F8	7	Factor VIII anti-hemophilic blood-clotting protein
	IL8	3	Interleukin 8 chemokine signaling protein
	KLRG1	8	Killer cell lectin-like receptor transmembrane protein
	LTA4H	9	Leukotriene A4 aminopeptidase bifunctional enzyme
	NLRP12	9	Cytoplasmic proteins involved in activation of inflammatory caspases
	PYDC1	13	Pyrin domain-containing protein 1, involved in suppression of kinase activity
	SLAMF7	6	Signaling lymphocytic activation molecule F7 immune cell receptor
	TLR1	11	Toll-like receptor 1, involved in identifying gram-positive bacterial infection
	TLR3.5	9	Toll-like receptor 3, involved in identifying viral infection

From Smith et al (2017).

Notes. CpG site IDs are available at Open Science Framework: https://osf.io/t4hkv/. To identify the probes, filter the table by gene name.

©2020 Reuben A et al. *JAMA Network Open*

eAppendix 4. Additional Details on the Selection of Probes for the Candidate Genes Analysis

We interrogated candidate genes hypothesized to be involved in inflammation and stress reactivity by identifying probes on the array that were annotated to prespecified genes in these domains. We chose probes annotated to these genes because they have been studied in the most detailed report about neighborhood disadvantage and DNA methylation.[6] Seven stress-reactivity-related genes and 11 inflammation-related genes were selected, with 3 to 66 probes annotated to each. Probe sequences for the Infinium HumanMethylation450 BeadChip kit were aligned to the hg19 version of the human genome using the BLAT[21] alignment algorithm. Probe sequences that mapped to multiple genomic loci were assigned to the genomic location provided by Illumina. Probe sequences that did not match any region of the genome with at least 94% identity were also assigned to the genomic location provided by Illumina. Each probe was then assigned to its nearest gene based on the GRCh37v75 ENSEMBL[22] release of the human transcriptome. For the purposes of this investigation, we also cross-referenced hg19 chromosomal locations for genes of interest with probe locations; this was to assign probes to the gene of interest in situations where multiple genes mapped proximally to the probe location.

eAppendix 5. Additional Details on the Creation of Polyepigenetic Scores (for Obesity, Inflammation, and Smoking) and the Measurement of Related Phenotypes

Obesity

Obesity Methylation Polygenic Score (ObPEGS). ObPEGS were calculated by averaging the product of CpG probe intensities in our data and estimated coefficients across each of 182 available CpG probes out of 187 identified as epigenome-wide significant in a meta-analysis of obesity.[23] To permit control for technical variation, variation due to blood cell composition and variation due to ethnicity, scores were residualised for methylation BeadChip-specific principal components (PCs), white blood cell counts and PCs computed from genome-wide SNP data. Scores were finally standardized to M=0 and SD=1.

The ObPEGS was validated in our study by correlating it with an obesity phenotype. Trained research workers took anthropometric measurements of Study members when they were aged 18 years. BMI was computed as weight in kilograms over squared height in meters. Waist\hip ratio was calculated by dividing waist circumference by hip circumference. We defined obesity using the US Centers for Disease Control and Prevention threshold of BMI>30 and the World Health Organization recommendation of waist-hip ratio >0.90 for men and >0.85 for women.[24] 21% of the analysis sample met at least one of these criteria, similar to prevalence for 16-24 year olds in the UK.[25]

Inflammation

Inflammation Methylation Polygenic Score (ImPEGS). ImPEGS were calculated by averaging the product of CpG probe intensities in our data and estimated coefficients across each of the 215 available CpG probes out of 218 identified as epigenome-wide significant in a meta-analysis of inflammation (C-reactive protein, CRP, level).[26] To permit control for technical variation, variation due to blood cell composition and variation due to ethnicity, scores were residualised for methylation BeadChip-specific PCs , white blood cell counts and PCs computed from genome-wide SNP data. Scores were finally standardized to M=0 and SD=1.

©2020 Reuben A et al. *JAMA Network Open*

The ImPEGS was validated in our study by correlating it with an inflammation phenotype (plasma C-reactive protein, CRP, level). Venous blood was collected from participants in EDTA tubes at age 18 years. Tubes were spun at 2,500 x g for 10 min, and plasma drawn off. Samples were stored at -80°C. Plasma was available for 1,448 participants. Plasma CRP (high-sensitivity CRP) was measured using Quantikine ELISA Kit DCRP00 (R&D Systems, Minneapolis, MN) following the manufacturer's protocol. The coefficient of variation (CV) was 5.6%. Plasma CRP level was log-transformed to improve normality of distribution, as is commonly done.[27] Further information on the inflammation phenotype is available in Rasmussen et al.

Smoking

Smoking Methylation Polygenic Score (SmPEGS). SmPEGS were constructed as described in Sugden et al.[29] Scores were calculated by averaging the product of CpG probe intensities in our data and estimated coefficients across each of the 2,480 available CpG probes out of 2,623 identified as epigenome-wide significant in a meta-analysis of current smoking.[30] To permit control for technical variation, variation due to blood cell composition and variation due to ethnicity, scores were residualised for methylation BeadChip-specific PCs, white blood cell counts and PCs computed from genome-wide SNP data. Scores were finally standardized to M=0 and SD=1.

The SmPEGS was validated in our study by correlating it with a smoking phenotype, assessed by self-reports of daily smoking behavior transformed into a continuous measure of pack-years-smoked based on the average number of cigarettes smoked a day multiplied by the number of years smoking by age 18.

eTable 4. Association Between Neighborhood Disadvantage and DNA Methylation

(A) shows the top 5 EWAS ‘hits’ with and without adjustment for tobacco smoking, family socioeconomic status, and maternal smoking while pregnant; (B) shows standardized effect sizes from OLS regression predicting methylation beta levels from neighborhood disadvantage using clustered error terms.

A

				**Association with neighborhood disadvantage without adjustment for pack-years-smoked**			**Association with neighborhood disadvantage with adjustment for pack-years-smoked**			**Association with neighborhood disadvantage with adjustment for family socioeconomic status**			**Association with neighborhood disadvantage with adjustment for maternal smoking while pregnant**	
**Probe ID**	**Gene Name**	**Position Relative To Gene**		**b**	***P***		**b**	***P***		**b**	***P***		**b**	***P***
cg25949550	CNTNAP2	INTRON		-9.61E-5	9.80E-12		-8.23E-5	1.82E-9		-5.18E-05	6.40E-04		-5.27E-5	1.05E-4
cg13570656	CYP1A1	Upstream		3.251E-4	9.42E-9		3.28E-4	1.20E-8		2.73E-04	3.57E-05		1.935E-4	1.07E-3
cg00213123	CYP1A1	Upstream		1.268E-4	6.58E-8		1.2825E-4	6.42E-8		9.96E-05	2.56E-04		7.39E-5	3.19E-3
cg17852385	CYP1A1	Upstream		2.008E-4	8.36E-8		2.018E-4	1.23E-7		1.69E-04	1.43E-04		1.247E-4	1.41E-3
cg12101586	CYP1A1	Upstream		2.948E-4	2.21E-7		3.042E-4	1.37E-7		2.66E-04	6.28E-05		1.764E-4	3.68E-3

©2020 Reuben A et al. *JAMA Network Open*

B

				Association with neighborhood disadvantage without adjustment for pack-years-smoked			Association with neighborhood disadvantage with adjustment for pack-years-smoked			Association with neighborhood disadvantage with adjustment for family socioeconomic status			Association with neighborhood disadvantage with adjustment for maternal smoking while pregnant	
**Probe ID**	**Gene Name**	**Position Relative To Gene**		**β**	**(95% CI)**		**β**	**95% CI**		**β**	**95% CI**		**β**	**95% CI**
cg25949550	CNTNAP2	INTRON		-.16	(-.20, -.11)		-.13	(-.17, -.09)		-.08	(-.13, -.04)		-.09	(-.13, -.05)
cg13570656	CYP1A1	Upstream		.14	(.09, .19)		.14	(.09, .19)		.11	(.05, .17)		.08	(.03, .13)
cg00213123	CYP1A1	Upstream		.13	(.08, .17)		.13	(.08, .17)		.09	(.04, .15)		.07	(.04, .12)
cg17852385	CYP1A1	Upstream		.13	(.08, .17)		.12	(.08, .17)		.10	(.05, .16)		.08	(.03, .13)
cg12101586	CYP1A1	Upstream		.13	(.08, .18)		.13	(.08, .18)		.11	(.05, .17)		.08	(.03, .13)

Notes. All associations are adjusted for the following additional covariates: sex, methylation-array control probe principal components indexing technical variation, and cell-type proportion estimates.

©2020 Reuben A et al. *JAMA Network Open*

eAppendix 6. Additional Details on the Measurement of Air Pollution Exposure

Exposure to nitrogen oxides (NO_x), a regulated gaseous pollutant composed of NO_2 and nitric oxide, and $PM_{2.5}$, a regulated aerosol pollutant with suspended solid and liquid particles smaller than 2.5 microns in diameter, was estimated for Study members at age 17 years based on pollution exposure estimates linked to the latitude-longitude coordinates of participants' residential addresses at 18 years of age (or where the participant spent most of their time) plus 2 additional addresses where the participants reported spending their time.[31] The most common locations were home, school, work, and shops. Creation of the pollution exposure estimates has been previously described [32]. Pollution exposure estimates were modeled using the local-scale Community Multiscale Air Quality (CMAQ-urban) Modeling System, which is a coupled regional chemical transport model and street-scale dispersion model. CMAQ-urban uses the newest generation of road traffic emissions inventory in the United Kingdom to model air quality down to individual streets, providing hourly estimates of pollutants at 20 × 20-m grid points throughout the United Kingdom (i.e., at address level). Study members' exposure to pollutants was estimated by calculating the mean levels of the pollutant across one year, 2012. Study member exposure to NO_x (mean annual level = 25.71 $\mu g/m^3$, SD = 16.28) fell within World Health Organization (WHO) guidelines for NO_2 (40 $\mu g/m^3$), a component of NO_x, while exposure to $PM_{2.5}$ (mean annual level = 11.24 $\mu g/m^3$, SD = 2.18) exceeded WHO guidelines (10 $\mu g/m^3$).[33] The two pollutants were highly correlated ($r=0.84$; $p< .001$).

eTable 5. Association of the Top 20 Neighborhood Disadvantage EWAS Probes With Estimates of Participants' Exposure to NO_x and $PM_{2.5}$

			Association with NO_x			Association with $PM_{2.5}$	
**Probe ID**	**Gene Name**		**β**	**(95% CI)**		**β**	**95% CI**
cg25949550	CNTNAP2		-.02	(-.07, .02)		.01	(-.04, .06)
cg13570656	CYP1A1		.06	(.00, .11)		.04	(-.01, .09)
cg00213123	CYP1A1		.03	(-.02, .08)		.04	(-.02, .09)
cg17852385	CYP1A1		.06	(.01, .11)		.01	(-.05, .07)
cg12101586	CYP1A1		.06	(.01, .11)		-.02	(-.08, .03)
cg16386523	OR4C13		.03	(-.03, .09)		.03	(-.02, .09)
cg07163531	PIGQ		-.01	(-.06, .05)		-.01	(-.06, .05)
cg22549041	CYP1A1		.05	(.00, .11)		.05	(.00, .11)
cg18474108	PABPC3		-.02	(-.08, .03)		.02	(-.04, .07)
cg03894796	RP13-82O9.5		.08	(.03, .12)		.07	(.03, .12)
cg05549655	CYP1A1		.07	(.02, .11)		.04	(-.01, .09)
cg06512316	RP13-7D7.1		.04	(-.02, .10)		.02	(-.03, .08)
cg18092474	CYP1A1		.09	(.04, .13)		.07	(.02, .12)
cg08529399	LA16c444G7.1		-.02	(-.08, .03)		-.01	(-.07, .04)
cg11775595	APC2		.07	(.01, .13)		.05	(-.01, .10)
cg15726514	C2orf43		.04	(-.02, .10)		.03	(-.02, .08)
cg16991589	PABPC3		-.06	(-.11, -.004)		-.01	(-.06, .04)
cg11640920	OR51I2		.06	(.01, .11)		.04	(-.01, .08)
cg11924019	CYP1A1		.08	(.02, .13)		.05	(.00, .10)
cg01145119	TOP1MT		.07	(.02, .12)		.05	(.00, .10)

Note. All associations are adjusted for sex, methylation-array control probe principal components indexing technical variation, and cell-type proportion estimates.

©2020 Reuben A et al. *JAMA Network Open*

eReferences

1. Coker ES, Gunier R, Huen K, Holland N, Eskenazi B. DNA methylation and socioeconomic status in a Mexican-American birth cohort. Clin Epigenetics [Internet]. 2018 [cited 2018 Oct 10];10. Available from: https://www.ncbi.nlm.nih.gov/pmc/articles/PMC5941629/

2. Janusek LW, Tell D, Gaylord-Harden N, Mathews HL. Relationship of childhood adversity and neighborhood violence to a proinflammatory phenotype in emerging adult African American men: An epigenetic link. Brain Behav Immun. 2017 Feb 1;60:126–35.

3. King KE, Kane JB, Scarbrough P, Hoyo C, Murphy SK. Neighborhood and family environment of expectant mothers may influence prenatal programming of adult cancer risk: discussion and an illustrative biomarker example. Biodemography Soc Biol. 2016;62(1):87–104.

4. Lei M-K, Beach SRH, Simons RL, Philibert RA. Neighborhood crime and depressive symptoms among African American women: Genetic moderation and epigenetic mediation of effects. Soc Sci Med. 2015 Dec 1;146:120–8.

5. Lei M-K, Simons RL, Beach SRH, Philibert RA. Neighborhood disadvantage and biological aging: Using marginal structural nodels to assess the link between neighborhood census variables and epigenetic aging. J Gerontol Ser B. 2019 Sep 15;74(7):e50–9.

6. Smith JA, Zhao W, Wang X, Ratliff SM, Mukherjee B, Kardia SLR, et al. Neighborhood characteristics influence DNA methylation of genes involved in stress response and inflammation: The Multi-Ethnic Study of Atherosclerosis. Epigenetics. 2017 Jul 5;12(8):662–73.

7. Wrigglesworth J, Ryan J, Vijayakumar N, Whittle S. Brain-derived neurotrophic factor DNA methylation mediates the association between neighborhood disadvantage and adolescent brain structure. Psychiatry Res Neuroimaging. 2019 Mar 30;285:51–7.

8. Giurgescu C, Nowak AL, Gillespie S, Nolan TS, Anderson CM, Ford JL, et al. Neighborhood Environment and DNA Methylation: Implications for Cardiovascular Disease Risk. J Urban Health. 2019 Mar 1;96(1):23–34.

9. Odgers CL, Caspi A, Russell MA, Sampson RJ, Arsenault L, Moffitt TE. Supportive parenting mediates widening neighborhood socioeconomic disparities in children’s antisocial behavior from ages 5 to 12. Dev Psychopathol. 2012 Aug;24(3):705–21.

10. Moffitt TE, E-Risk Study Team. Teen-aged mothers in contemporary Britain. J Child Psychol Psychiatry. 2002 Sep;43(6):727–42.

©2020 Reuben A et al. *JAMA Network Open*

11. Odgers CL, Caspi A, Bates CJ, Sampson RJ, Moffitt TE. Systematic social observation of children's neighborhoods using Google Street View: a reliable and cost-effective method. J Child Psychol Psychiatry. 2012 Oct;53(10):1009–17.

12. Sampson RJ, Raudenbush SW, Earls F. Neighborhoods and violent crime: a multilevel study of collective efficacy. Science. 1997 Aug 15;277(5328):918–24.

13. Sampson RJ, Morenoff JD, Earls F. Beyond social capital: Spatial dynamics of collective efficacy for children. Am Sociol Rev. 1999;64(5):633–60.

14. Marzi SJ, Sugden K, Arseneault L, Belsky DW, Burrage J, Corcoran DL, et al. Analysis of DNA methylation in young people: Limited evidence for an association between victimization stress and epigenetic variation in blood. Am J Psychiatry. 2018 Jun 1;175(6):517–29.

15. Pidsley R, Y Wong CC, Volta M, Lunnon K, Mill J, Schalkwyk LC. A data-driven approach to preprocessing Illumina 450K methylation array data. BMC Genomics. 2013 May 1;14:293.

16. Chen Y, Lemire M, Choufani S, Butcher DT, Grafodatskaya D, Zanke BW, et al. Discovery of cross-reactive probes and polymorphic CpGs in the Illumina Infinium HumanMethylation450 microarray. Epigenetics. 2013 Feb;8(2):203–9.

17. Price ME, Cotton AM, Lam LL, Farré P, Emberly E, Brown CJ, et al. Additional annotation enhances potential for biologically-relevant analysis of the Illumina Infinium HumanMethylation450 BeadChip array. Epigenetics Chromatin. 2013 Mar 3;6(1):4.

18. Houseman EA, Accomando WP, Koestler DC, Christensen BC, Marsit CJ, Nelson HH, et al. DNA methylation arrays as surrogate measures of cell mixture distribution. BMC Bioinformatics. 2012 May 8;13:86.

19. Horvath S. DNA methylation age of human tissues and cell types. Genome Biol. 2013 Dec 10;14(10):3156.

20. Cohen J. Statistical power analysis for the behavioral sciences. 2 edition. Hillsdale, N.J: Routledge; 1988. 400 p.

21. Kent WJ. BLAT—The BLAST-Like Alignment Tool. Genome Res. 2002 Apr;12(4):656–64.

22. Yates A, Akanni W, Amode MR, Barrell D, Billis K, Carvalho-Silva D, et al. Ensembl 2016. Nucleic Acids Res. 2016 Jan 4;44(Database issue):D710–6.

23. Wahl S, Drong A, Lehne B, Loh M, Scott WR, Kunze S, et al. Epigenome-wide association study of body mass index, and the adverse outcomes of adiposity. Nature. 2017 Jan;541(7635):81–6.

©2020 Reuben A et al. *JAMA Network Open*

24. WHO Expert Consultation. Waist circumference and waist–hip ratio: Report of a WHO Expert Consultation [Internet]. WHO. 2008 [cited 2016 Dec 5]. Available from: http://www.who.int/nutrition/publications/obesity/WHO_report_waistcircumference_and_waisthip_ratio/en/

25. Scantlebury R, Moody A. Health Survey for England, 2014, Chapter 9. Adult obesity and overweight [Internet]. The Health and Social Care Information Centre; 2014. (HSE). Report No.: Vol 1, Chapter 9. Available from: http://content.digital.nhs.uk/catalogue/PUB19295/HSE2014-ch9-adult-obe.pdf

26. Ligthart S, Marzi C, Aslibekyan S, Mendelson MM, Conneely KN, Tanaka T, et al. DNA methylation signatures of chronic low-grade inflammation are associated with complex diseases. Genome Biol. 2016 Dec 12;17(1):255.

27. The Emerging Risk Factors Collaboration. C-Reactive Protein, Fibrinogen, and Cardiovascular Disease Prediction. N Engl J Med. 2012 Oct 4;367(14):1310–20.

28. Rasmussen LJH. Improving the measurement of victimization-related inflammatory burden in young people: A longitudinal cohort study. JAMA Pediatr. 2019 in press;

29. Sugden K, Hannon EJ, Arseneault L, Belsky DW, Broadbent JM, Corcoran DL, et al. Establishing a generalized polyepigenetic biomarker for tobacco smoking. Transl Psychiatry. 2019 Feb 15;9(1):92.

30. Joehanes R, Just AC, Marioni RE, Pilling LC, Reynolds LM, Mandaviya PR, et al. Epigenetic signatures of cigarette smoking. Circ Cardiovasc Genet. 2016 Oct;9(5):436–47.

31. Newbury JB, Arseneault L, Beevers S, Kitwiroon N, Roberts S, Pariante CM, et al. Association of air pollution exposure with psychotic experiences during adolescence. JAMA Psychiatry. 2019;76(6):614-623.

32. Beevers SD, Kitwiroon N, Williams ML, Carslaw DC. One way coupling of CMAQ and a road source dispersion model for fine scale air pollution predictions. Atmospheric Environ Oxf Engl 1994. 2012 Nov;59(C):47–58.

33. World Health Organization. Air quality guidelines – global update 2005 [Internet]. Geneva, Switzerland; 2006 [cited 2019 Jun 11]. Available from: http://www.who.int/airpollution/publications/aqg2005/en/
